# Physicochemical characterization of etch‐and‐rinse adhesive system doped with catechin‐loaded polymeric microparticles

**DOI:** 10.1111/eos.70087

**Published:** 2026-03-12

**Authors:** Jiovanne Rabelo Neri, Nadine Luisa Guimarães Albuquerque, Marcelo Victor Sidou Lemos, Monica Yamauti, Francisco Fábio Oliveira de Sousa, Vanara Florêncio Passos, Sérgio Lima Santiago

**Affiliations:** ^1^ Graduate School of Dentistry University Center Christus Fortaleza Ceará Brazil; ^2^ Graduate Program in Dentistry Faculty of Pharmacy Dentistry and Nursing Federal University of Ceará Fortaleza Ceará Brazil; ^3^ School of Dentistry University of Fortaleza Fortaleza Ceará Brazil; ^4^ Department of Biomedical Sciences & Comprehensive Care Indiana University School of Dentistry Indianapolis Indiana USA; ^5^ Department of Pharmaceutical Sciences Federal University of Amapá Macapá Amapá Brazil

**Keywords:** catechin, dental adhesive, dentin, drug delivery systems, physical properties

## Abstract

This study evaluated the incorporation of epigallocatechin‐3‐gallate (EGCG)‐loaded poly(lactide‐*co*‐glycolide) (PLGA) microparticles into a two‐step etch‐and‐rinse adhesive and their effects on physicochemical properties and EGCG release. EGCG was added to Single Bond 2 either directly (0.01% and 0.1% w/w) or encapsulated in PLGA 50:50 or 75:25 microparticles (0.5%–2% w/w). Cumulative release was measured by UV–Vis spectrophotometry. Degree of conversion (DC) was analyzed by Fourier transform infrared spectroscopy; flexural strength and elastic modulus (E) were tested in three‐point bending; and water sorption (WS) and solubility (SL) were evaluated following ISO standards (*n* = 10). Microtensile bond strength (TBS) was tested after 24 h, 6, and 12 months. Data were analyzed by anova with significance set at *p* < 0.05. PLGA 50:50/EGCG at 1% showed the highest release, reaching 77.30 µg. No significant differences were found in DC, E, WS, and SL among the groups. Bond strengths remained stable in all experimental groups after 6 and 12 months, except for the control. Incorporating 1% EGCG‐loaded PLGA 50:50 microparticles into Single Bond 2 may represent a promising strategy for controlled release without compromising physicochemical or mechanical properties.

## INTRODUCTION

Biomodification of demineralized dentin matrices with cross‐linking agents has been investigated as a strategy to enhance the longevity of adhesive restorations [1, 2, 3, 4]. Such agents can improve the mechanical properties of collagen fibrils and reduce their enzymatic degradation by inhibiting matrix metalloproteinases (MMPs) and cysteine cathepsins (CTs) [1, 4, 5]. This may contribute to greater stability of the resin–dentin interface over time [2].

Bioactive agents in dentistry refer to substances or materials that interact with biological systems in a way that promotes beneficial effects, such as tissue regeneration. A large variety of bioactive agents can be found in the plants [1, 4]. Among the potential biomodifiers, epigallocatechin‐3‐gallate (EGCG), the main catechin of green tea, has received particular attention for its diverse therapeutic applications, ranging from antioxidant and anti‐inflammatory effects to potential anticancer properties. Moreover, EGCG has been reported to strengthen collagen through cross‐linking and to inhibit MMPs and CTs, thereby limiting enzymatic degradation of the hybrid layer [5, 6, 7, 8, 9]. Studies also indicate that EGCG may contribute to the preservation of resin–dentin bond strength [10, 11, 12]. However, when EGCG is directly incorporated into adhesives, its release is rapid and largely depleted within the first 24 h [13], raising concerns about its long‐term effectiveness.

Controlled release systems, such as poly(lactide‐*co*‐glycolide) (PLGA) microparticles, may overcome this limitation. PLGA is a biocompatible, biodegradable polymer widely used for drug delivery applications. Drug release from PLGA occurs through water penetration, polymer hydrolysis, and subsequent drug diffusion, with kinetics influenced by copolymer composition. Recent studies have shown that EGCG encapsulation in PLGA carriers exhibits improved stability, sustained release, and enhanced therapeutic efficacy [14, 15]. Key polymer characteristics, such as the lactic/glycolic acid ratio (e.g., 50:50 or 75:25), molecular weight, and structure configuration, can markedly affect the release profile by modulating degradation rate and diffusion pathways [16, 17, 18]. Despite these advantages, limited evidence exists on the impact of EGCG‐loaded PLGA microparticles on the performance of dental adhesives.

Therefore, this study aimed to evaluate the incorporation of EGCG‐loaded PLGA microparticles into a two‐step etch‐and‐rinse adhesive. The first null hypothesis was that no significant differences would be observed in EGCG release among the tested formulations. The second null hypothesis was that no significant differences would be observed in the physicochemical properties of the adhesive, regardless of the EGCG incorporation method.

## MATERIAL AND METHODS

### Materials

Two grades of PLGA were used: Resomer RG502H (PLGA 50:50, batch #STBD2887V) and Resomer RG756S (PLGA 75:25, batch #STBC6378V) (Sigma Aldrich, Germany). EGCG (batch #SLBL1959V) and ethyl acetate (batch #DCBB6676) were purchased from Sigma Aldrich (USA). Dichloromethane (DCM, batch #65456) was obtained from Dinâmica, Brazil. All reagents were of analytical grade and used without further purification.

### Synthesis of EGCG‐loaded PLGA microparticles

Microparticles were produced using a PLGA:EGCG mass ratio of 16:1. Two different forms of PLGA were used: 50:50 and 75:25, mentioned in materials. Due to differences in solubility (SL) among the drugs and polymer, an emulsification process was proposed. Briefly, PLGA 5.12% w/v dissolved in DCM and EGCG 0.64% dissolved in ethyl acetate were mixed under magnetic stirring for 10 min at 25°C using a high shear mixer (Ultra‐Turrax IKA T10B; IKA/Works) at 19,000 rpm for 5 min.

The resulting emulsion was spray‐dried using a Büchi B‐290 mini spray drier (Büchi Labortechnik AG), according to Sousa *et al.* [19]. The collected powders were transferred to glass vials and stored in desiccators at 4°C. A control formulation containing only PLGA was also prepared.

### Modification of the adhesive system

A commercial two‐step etch‐and‐rinse adhesive system, Adper Single Bond 2 (SB, 3 M ESPE), was used in this study (Table 1). EGCG was incorporated either directly or encapsulated within microparticles (Table 2). Modified adhesives were agitated in darkness for 1 min (tube shaker QL‐901, Biomixer) to ensure homogeneity. Only clear solutions with no visible crystals were used.

**TABLE 1:** Adhesive system and bonding procedures.

Product	Composition	Manufacturer (#Batch no.)	Application mode
Adper Single Bond 2	Adhesive—Bis‐GMA, HEMA, dimethacrylates, silica nanofiller (5 nm), polyalquenoic acid copolymer, initiators, water, and ethanol	3 M ESPE, St. Paul, MN, USA (batch #1312201025)	Two coats of adhesive Air‐drying for 10 s at 20 cm Light‐curing for 10 s

*Note*: This brand name is the same product as Adper Scotchbond 1 XT, Adper Single Bond Plus, and Adper Single Bond 1 XT.

Abbreviations: Bis‐GMA, bisphenol A diglycidyl methacrylate; HEMA, 2‐hydroxyethyl methacrylate.

**TABLE 2:** Experimental adhesives formulations and incorporation strategies of epigallocatechin‐3‐gallate (EGCG).

Adhesive formulations/groups	Incorporation mode	EGCG content (w/w%)	Description
Adper Single Bond 2 (SB)	–	–	Unmodified adhesive system
SB + 0.01% EGCG	Direct incorporation	0.01	Adhesive containing 0.01% EGCG
SB + 0.1% EGCG	Direct incorporation	0.1	Adhesive containing 0.1% EGCG
SB + 0.5% PLGA 50:50/EGCG	Microencapsulated (PLGA 50:50)	≈0.02	Adhesive containing 0.5% (w/w) PLGA 50:50 microparticles loaded with EGCG
SB + 1% PLGA 50:50/EGCG	Microencapsulated (PLGA 50:50)	≈0.04	Adhesive containing 1.0% (w/w) PLGA 50:50 microparticles loaded with EGCG
SB + 2% PLGA 50:50/EGCG	Microencapsulated (PLGA 50:50)	≈0.08	Adhesive containing 2.0% (w/w) PLGA 50:50 microparticles loaded with EGCG
SB + 0.5% PLGA75:25/EGCG	Microencapsulated (PLGA 75:25)	≈0.02	Adhesive containing 0.5% (w/w) PLGA 75:25 microparticles loaded with EGCG
SB + 1% PLGA75:25/EGCG	Microencapsulated (PLGA 75:25)	≈0.04	Adhesive containing 1.0% (w/w) PLGA 75:25 microparticles loaded with EGCG
SB + 2% PLGA75:25/EGCG	Microencapsulated (PLGA 75:25)	≈0.08	Adhesive containing 2.0% (w/w) PLGA 75:25 microparticles loaded with EGCG

Abbreviation: PLGA, poly(lactide‐*co*‐glycolide).

### Release assay of adhesives containing EGCG

To evaluate the influence of PLGA composition on EGCG release, an in vitro assay was performed. Standard solutions (2.5–40 µg/mL) of EGCG in distilled water were prepared to establish a calibration curve, which showed excellent linearity (*R* ^2^ = 0.99982). Measurements were performed at 275 nm using a UV–Vis spectrophotometer (DU‐730; Beckman Coulter).

Disc‐shaped specimens of adhesives (*n* = 9 per group; 6 mm × 1 mm) were fabricated using a silicone mold. Each disc was covered with a Mylar strip and glass slide, light‐cured for 20 s per side (VALO ULT7074B, Ultradent; 1000 mW/cm^2^), and polished to 0.5 mm thickness with 600‐grit SiC paper.

Each specimen was immersed in 1 mL distilled water at 37°C for up to 180 days (4320 h). At predetermined time points (0, 1, 3, 6, 12, 24, 48, 72, 96, 120, 240, 288, 336, 408, 466, 528, 576, 624, 672, 720, 768, 840, 888, 912, 984, 1032, 1128, 1200, 1344, 1608, 1848, 2064, 2424, 2904, and 4320 h), the storage medium was collected and analyzed by UV–Vis spectroscopy, and EGCG release was quantified using a calibration curve. Blank microparticles were also evaluated to correct for potential polymer interference. Based on the release profiles, the formulation exhibiting pulsatile release and the highest cumulative EGCG mass was selected for subsequent physicochemical characterization.

### Physicochemical characterization

Four adhesive formulations were tested: control (unmodified SB), 0.01% EGCG, 0.1% EGCG, and 1% PLGA/EGCG. The following properties were investigated: degree of conversion (DC), flexural strength (FS), elastic modulus (E), water sorption (WS), water SL, and microtensile bond strength (µTBS).

### Degree of conversion

DC was assessed by Fourier transform infrared spectroscopy (FTIR) (Perkin‐Elmer Spectrum 100, Perkin Elmer). Each adhesive was dispensed into a small agate mortar and thoroughly mixed with potassium bromide (KBr) using a pestle, at a ratio of 4:100 w/w. The pellets of KBr/adhesive solution were prepared with a hand press (Hand Press Kit 161‐1100, PIKE Technologies). FTIR spectrum of the uncured adhesive was obtained from each sample using 32 scans in a range of 4000–400 cm^−1^, at 4 cm^−1^ resolution in transmission mode.

The adhesive resins were light‐activated for 20 s using an LED light curing device at 1000 mW/cm^2^ irradiance (VALO ULT7074B, Ultradent Products). Additional FTIR spectra were obtained immediately after light curing. The analyses were performed at 25°C with 70% relative humidity. Ten specimens per group (*n * = 10) were tested. The rate of unreacted carbon–carbon double bonds (C = C) was determined from the ratio of absorbance intensities of aliphatic C = C (peak at 1636 cm^−1^) against an internal standard (aromatic carbon–carbon bond peak at 1608 cm^−1^) before and after curing. DC was determined by subtracting the C = C from 100%.

### Flexural strength and elastic modulus

FS and E were determined by a three‐point bending test following ISO 4049:2000, with adapted specimen dimensions (7 × 2 × 1 mm^3^) [20]. Specimens were fabricated in silicone molds‐10 uL of each adhesive formulation ‐ air‐dried (40 s), light‐cured (20 s per side), and stored in distilled water (37°C, 24 h). Testing was performed on a universal testing machine (Instron 3345) at 1 mm/min. FS and E were calculated from specimen dimensions measured with a digital caliper (Mitutoyo).

### Water sorption/solubility

WS and SL were evaluated according to ISO 4049:2000 with modified dimensions [19].

Ten disc specimens (6.0 mm diameter and 1.0 mm thickness) were prepared as previously described in EGCG release test. The discs were stored in a silica‐containing desiccator at 37°C and were repeatedly weighed after 24‐h intervals on an analytical balance (AUX‐220, Shimadzu, Tokyo, Japan) with an accuracy of 0.0001 g up to a constant mass (*m*1) was obtained (i.e., variation less than 0.1 mg in three weight measures), after 2 days. The volume of each specimen was measured with a 0.001 mm precision digital caliper (Absolute Digimatic, Mitutoyo) by analyzing the diameter and thickness, and the volume (V) was expressed in mm^3^. Thereafter, the specimens were stored in sealed glass vials with 1.5 mL of distilled water at 37°C for 7 days. Afterward, the specimens were weighed after gently wiped on absorbent papers to obtain a constant mass (*m*2), and then they were returned to the desiccator. The specimens were finally weighed as aforementioned up to stabilization of a constant mass (*m*3), after 3 days. WS and SL (µg/mm^3^) were calculated using the following formulae:

$$\mathrm{WS}=\frac{m2-m3}{V}\;\mathrm{SL}=\frac{m1-m3}{V}$$

### Microtensile bond strength test (μεTBS)

Thirty‐six unerupted, caries‐free third molars were collected after the patients' informed consent had been obtained under a protocol reviewed and approved by the local Research and Ethics Committee (#459.659). Teeth were stored in 0.01% thymol and used within 1 month. After occlusal enamel removal and dentin surface preparation with #600 SiC paper, teeth were randomly assigned to four groups (*n* = 9) by the Excel software (Excel 2013, Microsoft Corporation, One Microsoft Way). The dentin was conditioned with 35% phosphoric acid, rinsed, and excess water was carefully removed with absorbent paper, keeping the substrate moist. Adhesive was applied according to manufacturer's instructions (Table 1), followed by incremental build‐up of composite resin (Filtek Z250XT, 3 M ESPE).

After 24 h storage, specimens were sectioned to obtain bonded sticks (∼1.0 mm^2^ cross‐sectional area). Sticks were tested after 24 h, 6 months, or 12 months of water storage (37°C, solution refreshed biweekly). Specimens were fixed to a testing jig with cyanoacrylate and loaded in tension at 0.5 mm/min (Emic). Bond strength (MPa) was calculated from failure load and cross‐sectional area. Failure modes were assessed under stereomicroscopy (Leica S8 APO) and categorized as adhesive, cohesive (in dentin or resin), or mixed. Representative specimens were sputter‐coated with gold‐palladium and observed under scanning electron micrograph (SEM) (Quanta FEG 450, FEI).

### Statistical analysis

Statistical procedures were performed with the Sigma Plot 14.0 software (SYSTAT) for Windows statistical program software. A Shapiro–Wilk test was applied to all groups to analyze the normal distribution of errors and the Bartlett test for the homoscedasticity. Cumulative EGCG release in 24 and 4320 h was analyzed by two‐way anova on ranks (factors: storage time and adhesive system) with Holm–Sidak post hoc tests. One‐way anova was used to compare DC, FS, E, WS and SL. µTBS was analyzed by two‐way anova, followed by Student Newman Keuls tests. The significance level was set at *p* < 0.05. Teeth were used as a statistical unit, and the number of prematurely debonded specimens was recorded but excluded from the analysis.

## RESULTS

EGCG release profiles are shown in Figures 2 and 3. Cumulative EGCG release (%) was significantly influenced by adhesive formulation (*p* < 0.001; *F* = 9.399) and storage time (*p* < 0.001; *F* = 140.924). The interactions between variables were significant (*p* < 0.001; *F* = 5.909). At 24 h, no significant difference was observed among the groups (*p* > 0.05). After 4320 h, there was no significant difference among the 0.5% PLGA 50:50/EGCG, 1% PLGA 50:50/EGCG, and 0.01% EGCG (*p* > 0.05), all of which reached complete release (100%) during the assay period. On the other hand, the PLGA75:25 groups exhibited incomplete release, with the highest value not exceeding 68% of the incorporated drug content (0.5% PLGA75:25/EGCG).

**FIGURE 1:**
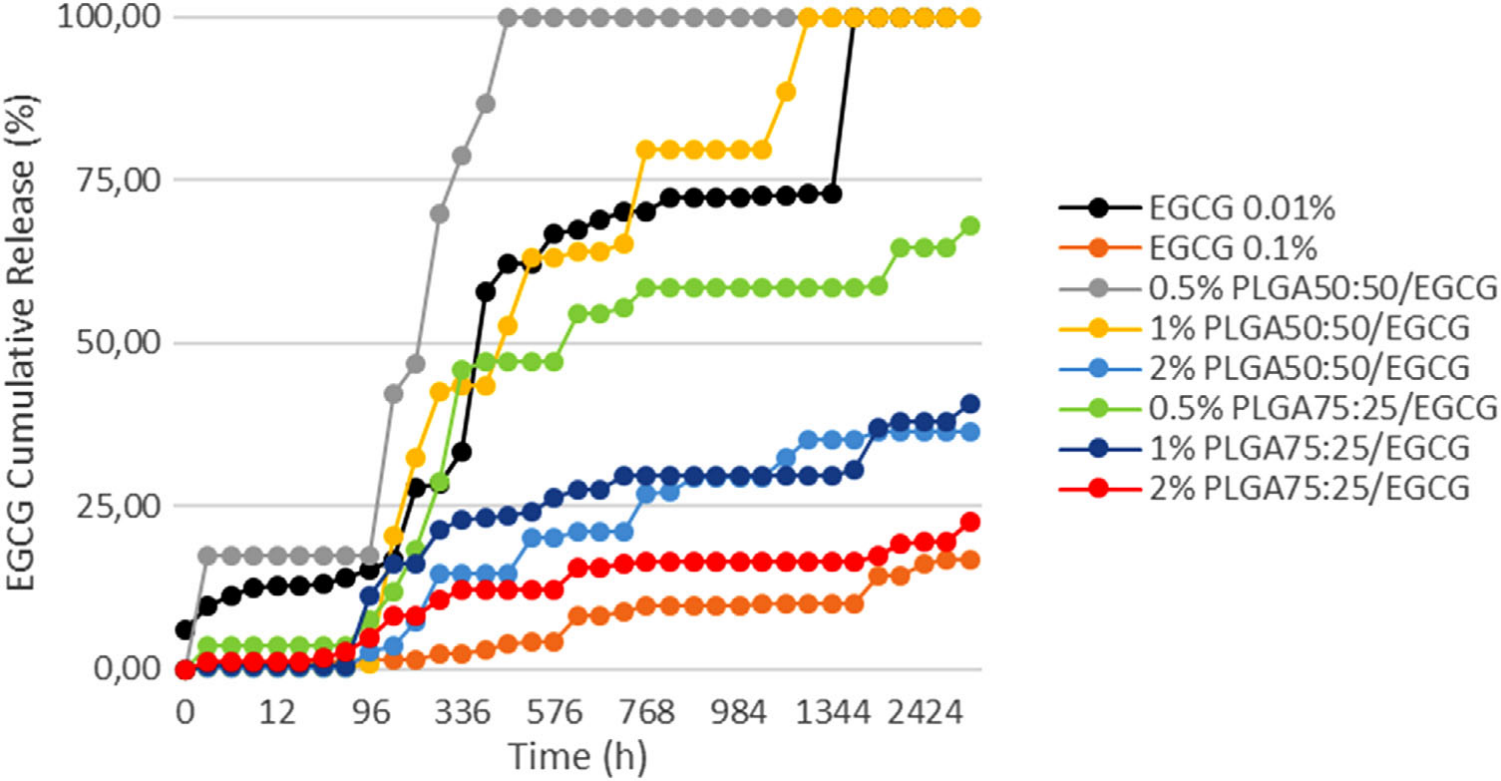
Epigallocatechin‐3‐gallate (EGCG) cumulative release (%) from adhesive systems during the entire evaluation period (4320 h). PLGA, poly(lactide‐*co*‐glycolide).

**FIGURE 2:**
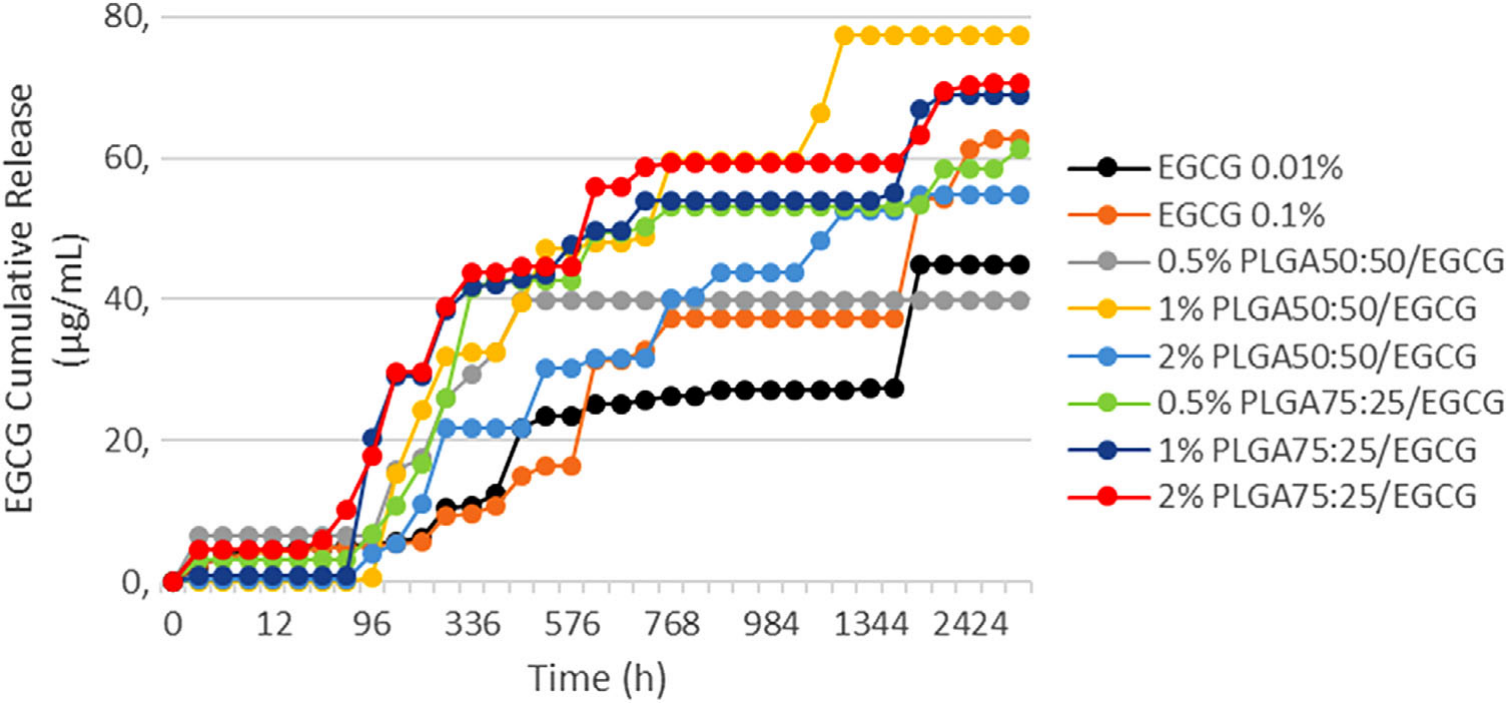
Epigallocatechin‐3‐gallate (EGCG) cumulative release (µg/mL) from adhesive systems during the entire evaluation period (4320 h). PLGA, poly(lactide‐*co*‐glycolide).

All groups containing PLGA microparticles showed a pulsatile release profile, characterized by alternating phases of latency and accelerated release. The 1% PLGA 50:50/EGCG group presented a four‐step burst release pattern, reaching 42.62% at 288 h, 63% at 528 h, 79.67% at 768 h, and 100% at 1200 h (Figure 1). Moreover, this group achieved the highest quantitative release, with a total released mass of 77.30 µg completing the full release within 50 days (Figure 2). By comparison, the 0.5% PLGA 50:50/EGCG group showed the lowest release (39.84 µg) (Figure 2). Based on these findings, the 1% PLGA 50:50/EGCG was selected for subsequent analyses of physicochemical and mechanical properties.

Table 3 presents the mean values and standard deviation of DC, FS, E, WS, and SL for the tested adhesive formulation. No statistically significant difference was found among the groups for DC, E, WS, and SL (*p* > 0.05). However, FS was influenced by adhesive formulation (*p* = 0.009; *F* = 4.432). The incorporation of 1% PLGA 50:50/EGCG resulted in a significant increase in FS (*p* < 0.05), whereas no statistically significant difference was observed among the other groups. (*p* > 0.05). Mean µTBS values are shown in Table 4. µTBS was significantly influenced by the adhesive formulation (*p* < 0.001; *F* = 7.319) and storage time (*p* < 0.001; *F* = 10.555). No statistically significant interaction between these factors was observed (*p* = 0.45; *F* = 0.905). After 24 h of storage, there were no significant differences in mean bond strength among the groups (*p* > 0.05). At 6 months, no statistical difference was found among the EGCG groups or between 1% PLGA 50:50/EGCG and SB (*p* > 0.05). At 12 months, all EGCG groups presented significantly higher values than the SB group (*p* < 0.05). Furthermore, resin–dentin bond strength was preserved at 6 and 12 months in both the direct and microencapsulated EGCG groups (*p* > 0.05).

**TABLE 3:** Mean (standard deviation) of physicochemical properties tested.

Groups (*n *= 10)	Degree of conversion %	Flexural strength (MPa)	Elastic modulus (GPa)	Water sorption (µg/mm^3^)	Solubility (µg/mm^3^)
SB (control)	59.8 (2.0)	12.2 (3.1)^a^	0.2 (0.06)	172.7 (7.2)	73.4 (5.8)
0.01% EGCG	58.3 (3.3)	12.4 (2.7)^a^	0.2 (0.1)	175.5 (6.3)	74.7 (2.8)
0.1% EGCG	59.0 (3.6)	11.9 (3.7)^a^	0.2 (0.09)	173.6 (9.6)	76.3 (5.2)
1% PLGA 50:50/EGCG	58.7 (2.8)	16.5 (3.4)^b^	0.2 (0.04)	166.9 (7.4)	77.0 (4.3)

*Note*: Distinct superscript letters (^a,b^) indicate statistical difference in the same columns (*p* < 0.05).

Abbreviations: EGCG, epigallocatechin‐3‐gallate; PLGA, poly(lactide‐*co*‐glycolide).

**TABLE 4:** Bond strength values [MPa ± SD (*)] according to adhesive systems used.

Groups (*n *= 9)	Adper Single Bond 2		
	24 h (ns)	6 months	12 months
SB	34.6 ± 6.9 (47)^A,a^	26.5 ± 6.5 (50)^A,b^	23.5 ± 2.1 (45)^A,b^
0.01% EGCG	36.6 ± 5.7 (53)^A,a^	34.6 ± 4.2 (46)^B,a^	32.4 ± 6.2 (58)^B,a^
0.1% EGCG	38.6 ± 4.7 (52)^A,a^	35.7 ± 6.7 (51)^B,a^	32.4 ± 5.9 (59)^B,a^
1% PLGA 50:50/EGCG	35.1 ± 6.7 (40)^A,a^	29.6 ± 9.3 (35)^AB,a^	30.2 ± 6.2 (34)^B,a^

*Note*: Identical superscript letters indicate no statistical significance between values. Capital letters compare treatments, and lowercases compare storage time. (*) Corresponds to the number of sticks tested per group in each period.

Abbreviations: EGCG, epigallocatechin‐3‐gallate; PLGA, poly(lactide‐*co*‐glycolide).

Table 5 shows the distribution of failure modes at 24 h and 6 and 12 months. At all time points, most failures were mixed in all tested groups, except for the SB group at 12 months, in which adhesive failures were most prevalent. Adhesive failures increased in all groups, particularly between 24 h and 6 months. Cohesive failures (in both resin and dentin) also increased over time in all groups, except for the 1% PLGA 50:50/EGCG group, which showed a decrease between 24 h and 6 months. Premature failures increased in all groups from 24 h to 6 months. However, between 6 and 12 months, premature failures remained stable in the SB and 1% PLGA 50:50/EGCG groups, whereas a reduction was observed in the EGCG 0.01% and EGCG 0.1% groups.

**TABLE 5:** Distribution of mode of fracture of each group expressed as *n* (relative percentage).

Groups	Adper Single Bond 2														
	24 h					6 months					12 months				
	A	M	CR	CD	PF	A	M	CR	CD	PF	A	M	CR	CD	PF
SB	7 (14)	40 (78)	1 (2)	2 (4)	1 (2)	16 (26)	34 (55)	4 (6)	3 (5)	5 (8)	28 (41)	17 (25)	7 (10)	11 (16)	6 (8)
EGCG 0.01%	1 (2)	52 (88)	3 (5)	1 (2)	2 (3)	7 (12)	39 (65)	6 (10)	5 (8)	3 (5)	8 (11)	50 (72)	5 (7)	4 (6)	3 (4)
EGCG 0.1%	3 (5)	49 (82)	3 (5)	3 (5)	2 (3)	6 (9)	45 (65)	10 (15)	3 (4)	5 (7)	13 (14)	46 (50)	17 (19)	13 (14)	3 (3)
1% PLGA 50:50/EGCG	2 (4)	38 (81)	3 (7)	2 (4)	2 (4)	7 (18)	28 (74)	1 (3)	0 (0)	2 (5)	6 (15)	28 (68)	4 (10)	1 (2)	2 (5)

Abbreviations: A, adhesive failure; CD, cohesive failure in dentin; CR, cohesive failure in resin; M, mixed failure; PF, premature failure.

**FIGURE 3:**
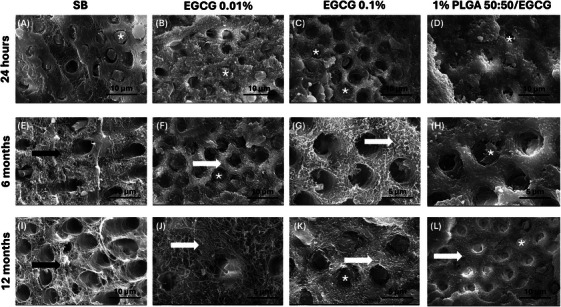
Representative scanning electron micrographs (SEM) of the dentin side of fractured specimens from SB, epigallocatechin‐3‐gallate (EGCG) 0.01%, EGCG 0.1%, and 1% poly(lactide‐*co*‐glycolide) (PLGA) 50:50/EGCG groups after 24 h (A–D), 6 months (E–H), and 12 months (I–L) of storage. Original magnifications were 5000× for parts (A–H), 10,000× for parts (I, K, and L), and 30,000× for part (J). Asterisks (*) indicate the presence of resin tags. Preserved collagen fibrils in intertubular dentin are indicated by white arrows, whereas collagen fibrils undergoing degradation are indicated by black arrows.

SEM analysis revealed marked differences in the morphology of the adhesive interface among the experimental groups over time (Figure 3). At 24 h, all groups exhibited well‐defined resin tags penetrating into the dentinal tubules (*), with no evident degradation of the collagen network. After 6 months, the control group (SB) showed pronounced collagen degradation in the intertubular dentin (black arrow), whereas the EGCG‐treated groups demonstrated better preservation of fibrillar organization. This effect was particularly evident in the 0.1% EGCG group, where collagen fibrils remained clearly identifiable (white arrow). In contrast, the PLGA/EGCG group exhibited both intact resin tags and preserved collagen fibrils, suggesting a sustained protective effect. At 12 months, the SB group showed extensive collagen breakdown with loss of structural integrity (black arrow), whereas the EGCG‐containing groups retained more organized collagen fibrils (white arrow). The preservation was most notable in the 0.1% EGCG and PLGA/EGCG formulations, in which dentinal tubules remained discernible and intertubular collagen appeared stabilized.

## DISCUSSION

The use of adhesive systems as carriers for therapeutic agents is an attractive strategy to improve the longevity of resin–dentin interfaces [20]. Several bioactive compounds have been incorporated into adhesives in an attempt to preserve bond durability both in vitro [3, 6, 9, 12, 21, 22, 23, 24] and in vivo [25, 26, 27, 28]. However, the interaction of bioactive molecules with resin monomers may influence fundamental properties such as DC, crosslinking density, mechanical strength, WS, and release profile [7, 8, 9, 10, 11, 12, 13].

For enzymatic inhibition to occur, adhesive systems must release EGCG at concentrations above the inhibitory thresholds. EGCG has been reported to inhibit MMP‐2 and MMP‐9 at 6 µM (≈20 µg/mL) and 0.8 µM (≈3 µg/mL), respectively [29, 30], whereas CT‐B inhibition requires much higher concentrations (≈6500 µg/mL) [4]. After 4320 h in distilled water, all experimental groups released sufficient EGCG to potentially inhibit MMPs, but not CTs. Considering that fluid within the hybrid layer is limited (≈4 µL/cm^2^/min) [31], local EGCG concentrations at the interface may be higher than those measured in our release assay, which represents a limitation of this methodology.

In this study, PLGA microparticles were designed to act as temporary carriers for controlled EGCG release rather than as structural components of the hybrid layer. The low incorporation level (1 wt%) is unlikely to disrupt the adhesive polymer network or induce clinically relevant mechanical compromise.

The release assays demonstrated distinct patterns depending on the mode of incorporation. Direct addition of EGCG at 0.01% and 0.1% resulted in low and constant release profile, in agreement with previous studies [13]. Although this pattern may be insufficient to fully inhibit collagenolytic enzymes, some authors have reported preservation of bond strength over 6 months with low concentrations [6], suggesting that even minimal release may exert a protective effect. By contrast, PLGA microparticles promoted a pulsatile release profile, potentially allowing periodic attainment of inhibitory concentrations. As a biodegradable polymer with tunable degradation kinetics, PLGA enables predictable and sustained drug delivery [16, 32, 33, 34]. Pulsatile release enables intermittent higher doses [35], which may be advantageous for inactivating dentin endopeptidases that degrade the hybrid layer. Importantly, PLGA biodegradation should not be interpreted as a source of clinical instability, but rather as a mechanism supporting sustained EGCG availability. Moreover, conventional adhesive monomers such as HEMA, Bis‐GMA, and TEGDMA are also susceptible to hydrolytic degradation and remain clinically accepted. Therefore, degradability alone does not imply material compromise; concentration and functional role within the system must be considered. The present outcome revealed that after 4320 h, PLGA 50:50 exhibited higher cumulative release than PLGA 75:25, confirming the influence of copolymer composition on release kinetics and leading to rejection of the first null hypothesis.

With regard to mechanical properties, DC was not affected by EGCG, either in free form or when encapsulated. This corroborates earlier reports that low concentrations of catechins do not interfere with polymerization [6, 7, 9, 13, 21, 24], possibly because the molecules are entrapped in the polymeric network after curing without disturbing monomer conversion [7]. It should be noted that the degree‐of‐conversion analysis was performed using FTIR with KBr, which may be considered a methodological limitation and should be taken into account when interpreting the results. The lack of effect from PLGA microparticles further suggests chemical compatibility between the adhesive monomers and the biodegradable polymer [16]. Interestingly, FS values increased when EGCG was incorporated via PLGA microparticles, which may be explained by undissolved particles acting as reinforcing fillers within the resin matrix [36]. Accordingly, the second null hypothesis was also rejected.

The evaluation of WS and SL provided further insights into the interaction between EGCG and the adhesive matrix. Simplified adhesives are known for their hydrophilic composition and susceptibility to sorption and SL [37, 38, 39], as also confirmed for Adper Single Bond 2 in previous studies [40, 41, 42]. Despite EGCG's hydrophilic nature and high potential for hydrogen bonding, its incorporation at the tested concentrations did not increase water uptake. This outcome may be related to the relatively low amounts used and the hydrophobic character of PLGA, which resists water penetration [43]. Similar findings were reported by Pallan *et al.* [13], who observed no significant changes in WS with EGCG up to 2%. WS is directly related to SL, characterized by leaching of solvents, unreacted monomers, and oligomers [39]. In the literature, conflicting SL results have been reported for adhesives containing EGCG. Pallan *et al.* [13] observed a concentration‐dependent increase in SL, whereas Neri *et al.* [7] reported decreased SL in a one‐step self‐etch adhesive doped with 0.01% and 0.1% EGCG. In the present study, however, EGCG incorporation—whether direct or via PLGA microparticles—did not significantly affect SL values of the two‐step etch‐and‐rinse adhesive.

Although no significant differences in water SL were observed among the experimental groups, distinct EGCG release profiles were detected. This apparent discrepancy can be explained by differences in release mechanisms rather than bulk material dissolution. Water SL primarily reflects the overall mass loss of the material when exposed to an aqueous environment, whereas EGCG release is governed by diffusion processes, polymer–drug interactions, and the microstructure of the polymeric/resin matrix. In the present study, the incorporation of EGCG or PLGA/EGCG did not compromise the integrity or SL of the matrix; however, it modulated the mobility and availability of EGCG within the material. The pulsatile release behavior observed may be associated with localized diffusion pathways, gradual polymer relaxation, and controlled degradation of PLGA microparticles, which allow EGCG to be released without inducing measurable changes in bulk SL. These findings indicate that variations in release kinetics can occur independently of water SL, supporting the absence of significant differences in SL despite distinct release profiles.

Immediate bond strength depends on the adequate infiltration of resin monomers into the interfibrillar spaces of demineralized dentin, leading to hybrid layer formation. However, differences in adhesive composition may influence this process [1, 44, 45]. In the present study, the incorporation of EGCG into the adhesive system did not affect resin–dentin bond strength after 24 h. These outcomes are consistent with the distribution of fracture modes, as a high percentage of mixed failures was observed across all groups (Table 5), a trend also reported by Guimarães *et al.* [9]. Furthermore, SEM micrographs revealed similar patterns of hybrid layer formation and effective sealing of dentinal tubules in all groups, confirming proper initial adaptation of the adhesive system (Figure 3A–D).

Importantly, our results demonstrated that EGCG exerted a positive effect on bond strength after both 6 and 12 months of water storage. At 6 months, higher bond strength values were predominantly observed for EGCG concentrations directly incorporated into the adhesive, whereas at 12 months, both incorporation strategies proved effective. A statistically significant difference was observed compared with the control group, in line with previous reports [6, 9, 12, 21, 24, 34]. EGCG is known to interact with collagen fibrils, promote cross‐linking, and reduce the enzymatic activity of MMPs and CTs—mechanisms that may contribute to the long‐term stability of adhesive interfaces [4]. SEM analysis supported these findings: after 6 and 12 months, the EGCG‐containing groups showed superior preservation of the collagen matrix compared with the control (Figure 3E,I). At 6 months, collagen fibrils appeared more organized, with reduced structural collapse and only moderate exposure of the underlying collagen, indicating a protective effect against enzymatic degradation (Figure 3F–H). At 12 months, although further deterioration was evident, the structural integrity of the EGCG groups remained superior to that of the conventional adhesive, with discernible dentinal tubules and preserved fibrillar organization, particularly at the 0.1% concentration (Figure 3J–L). Collectively, these findings suggest that EGCG contributes to hybrid layer stabilization and delays adhesive interface degradation over time. Longer evaluation periods, particularly in caries‐affected dentin, are still needed to confirm these effects under clinically challenging conditions.

The distinct bond strength behavior observed over time for directly incorporated and PLGA‐encapsulated EGCG can be attributed to differences in their release kinetics. Although EGCG directly incorporated into polymeric materials is rapidly released, as demonstrated by Pallan *et al.* [13], its early availability may be sufficient to promote initial collagen cross‐linking and transient inhibition of MMPs and CTs. This mechanism may explain the improved bond strength observed at intermediate storage periods, such as 6 months. In contrast, EGCG encapsulated within PLGA microparticles is released in a delayed and more sustained manner, which may limit its immediate bioavailability but ensures prolonged enzymatic inhibition and collagen stabilization. This sustained effect becomes particularly relevant at longer storage times, such as 12 months, when degradation of the hybrid layer is more pronounced. Accordingly, free and encapsulated EGCG appear to operate within distinct temporal windows, with direct incorporation favoring short‐ to mid‐term preservation of bond strength, whereas PLGA‐mediated delivery contributes more effectively to long‐term stability. Collectively, these findings underscore the role of controlled‐release systems not in enhancing immediate adhesive performance, but in extending the protective effects of bioactive agents over time, thereby addressing the limitations associated with the rapid depletion of EGCG when directly incorporated into adhesive formulations.

Based on the overall findings of this study, the adhesive formulation consisting of Single Bond 2 doped with 1 wt% EGCG‐loaded PLGA 50:50 microparticles appears to be the most promising for future investigations. This formulation demonstrated a controlled EGCG release profile while preserving the physicochemical properties of the adhesive system. The combination of sustained bioactive agent delivery and maintained material performance suggests a favorable balance between biological modulation and material stability. Collectively, these results indicate that this formulation may contribute to improved long‐term resin–dentin bond durability and support further exploration of controlled‐release strategies as a potential paradigm shift in adhesive dentistry.

## AUTHOR CONTRIBUTIONS

**Conceptualization**: Jiovanne Rabelo Neri, Sérgio Lima Santiago. **Formal analysis**: Marcelo Victor Sidou Lemos, Vanara Florêncio Passos. **Methodology**: Jiovanne Rabelo Neri, Nadine Luisa Guimarães Albuquerque. **Validation**: Monica Yamauti. **Writing—original draft**: Jiovanne Rabelo Neri, Nadine Luisa Guimarães Albuquerque. **Writing—review and editing**: Sérgio Lima Santiago, Francisco Fábio Oliveira de Sousa, Monica Yamauti. **Resources**: Sérgio Lima Santiago. **Supervision**: Francisco Fábio Oliveira de Sousa, Vanara Florêncio Passos, Sérgio Lima Santiago. **Investigation**: Nadine Luisa Guimarães Albuquerque.

## CONFLICT OF INTEREST STATEMENT

The authors declare no conflicts of interest.

## DECLARATION OF GENERATIVE AI AND AI‐ASSISTED TECHNOLOGIES IN THE WRITING PROCESS

During the preparation of this work, the authors used ChatGPT Plus (OPEN AI) in order to review text fluency and language, spelling, and grammar errors. This tool was not used to generate content. After using this service, the authors reviewed and edited the content as needed and take full responsibility for the content of the publication.

## References

[1] Breschi L , Maravic T , Mazzitelli C , Josic U , Mancuso E , Cadenaro M , et al. The evolution of adhesive dentistry: from etch‐and‐rinse to universal bonding systems. Dent Mater. 2025;41:141–58. 39632207 10.1016/j.dental.2024.11.011

[2] Bedran‐Russo AK , Pauli GF , Chen SN , McAlpine J , Castellan CS , Phansalkar RS , et al. Dentin biomodification: strategies, renewable resources and clinical applications. Dent Mater. 2014;30:62–76. 24309436 10.1016/j.dental.2013.10.012 PMC3972923

[3] Santiago SL , Osorio R , Neri JR , Carvalho RM , Toledano M . Effect of the flavonoid epigallocatechin‐3‐gallate on resin‐dentin bond strength. J Adhes Dent. 2013;15:535–40. 23560257 10.3290/j.jad.a29532

[4] Vidal CM , Aguiar TR , Phansalkar R , McAlpine JB , Napolitano JG , Chen SN , et al. Galloyl moieties enhance the dentin biomodification potential of plant‐derived catechins. Acta Biomater. 2014;10:3288–94. 24721612 10.1016/j.actbio.2014.03.036 PMC4041811

[5] Epasinghe DJ , Yiu CK , Burrow MF , Tsoi JK , Tay FR . Effect of flavonoids on the mechanical properties of demineralised dentine. J Dent. 2014;42:1178–84. 25010542 10.1016/j.jdent.2014.07.002

[6] Du X , Huang X , Huang C , Wang Y , Zhang Y . Epigallocatechin‐3‐gallate (EGCG) enhances the therapeutic activity of a dental adhesive. J Dent. 2012;40:485–92. 22421091 10.1016/j.jdent.2012.02.013

[7] Neri JR , Yamauti M , Feitosa VP , Pires AP , Araújo RS , Santiago SL . Physicochemical properties of a methacrylate‐based dental adhesive incorporated with epigallocatechin‐3‐gallate. Braz Dent J. 2014;25:528–31. 25590200 10.1590/0103-6440201300096

[8] Mendes TAD , Pascoal SCD , Estellita MCA , Lemos MVS , Santiago SL , Mendonça JS . Chemical analysis of n‐propil gallate used as pretreatment for resin‐dentin bond strength: in vitro study. Eur J Oral Sci. 2024;132:e12970. 38173083 10.1111/eos.12970

[9] Guimarães NLA , Costa CA , Neri JR , Mota ALM , Lemos MVS , Mendonça JS , et al. Catechin‐doped universal adhesive system: physicochemical characterization and resin‐dentin bonding stability. Int J Adhesion Adhes. 2025;140:1–8.

[10] Chen H , Huang B . Effect of EGCG application on collagen degradation in dentine caries. Appl Mech Mater. 2013;455:112–6.

[11] Jackson JK , Zhao J , Wong W , Burt HM . The inhibition of collagenase induced degradation of collagen by the galloyl‐containing polyphenols tannic acid, epigallocatechingallate and epicatechingallate. J Mater Sci‐Mater M. 2010;21:1435–43. 20162329 10.1007/s10856-010-4019-3

[12] Neri JR , Yamauti M , Silveira, FD , Mendonça JS , Carvalho RM , Santiago SL . Influence of dentin biomodification with epigallocatechin‐3‐gallate on the bond strength of self‐etch adhesive: twelve‐month results. Int J Adhesion Adhes. 2016;71:81–6.

[13] Pallan S , Furtado Araujo MV , Cilli R , Prakki A . Mechanical properties and characteristics of developmental copolymers incorporating catechin or chlorhexidine. Dent Mater. 2012;28:687–94. 22460187 10.1016/j.dental.2012.03.003

[14] Minnelli C , Stipa P , Sabbatini S , Mengucci P , Mobbili G , Galeazzi R , et al. Insights into PLGA‐encapsulated epigallocatechin 3‐gallate nanoparticles as a new potential biomedical system: a computational and experimental approach. Eur Polym J. 2023;182:111723.

[15] Wong CN , Lim YM , Liew KB , Chew Y‐L , Chua A‐L . EGCG as a therapeutic agent: a systematic review of recent advances and challenges in nanocarrier strategies. J Zhejiang Univ‐Sci B (Biomed & Biotechnol). 2025;26:633–56. 10.1631/jzus.B2400040 PMC12303791 40722243

[16] Matsumoto A , Matsukawa Y , Suzuki T , Yoshino H . Drug release characteristics of multi‐reservoir type microspheres with poly(dl‐lactide‐co‐glycolide) and poly(dl‐lactide). J Control Release. 2005;106:172–80. 15936109 10.1016/j.jconrel.2005.03.026

[17] Li M , Rouaud O , Poncelet D . Microencapsulation by solvent evaporation: state of the art for process engineering approaches. Int J Pharm. 2008;363:26–39. 18706988 10.1016/j.ijpharm.2008.07.018

[18] Makadia HK , Siegel SJ . Poly lactic‐co‐glycolic acid (PLGA) as biodegradable controlled drug delivery carrier. Polymers (Basel). 2011;3:1377–97. 22577513 10.3390/polym3031377 PMC3347861

[19] Sousa FFO , Blanco‐Mendez J , Perez‐Estevez A , Seoane‐Prado R , Luzardo‐Alvarez A . Effect of zein onbiodegradable inserts for the delivery of tetracycline within periodontal pockets. J Biomater Appl. 2012;27:187–200. 21586598 10.1177/0885328211398968

[20] Cadenaro M , Pashley DH , Marchesi G , Carrilho M , Antoniolli F , Mazzoni A , et al. Influence of chlorhexidine on the degree of conversion and E‐modulus of experimental adhesive blends. Dent Mater. 2009;25:1269–74. 19570568 10.1016/j.dental.2009.05.008

[21] Sousa NO , Maia SJM , Lima KER , Moreira MM , Rifane TO , Saboia VPA , et al. Incorporation of epigallocatechin‐3‐gallate absorbed onto hydroxyapatite nanoparticles in dental adhesive: Impact on resin dentin‐bonding, chemical, and mechanical properties Int J Adhesion Adhes. 2025;140:104019.

[22] Carrilho MR , Carvalho RM , de Goes MF , di Hipólito V , Geraldeli S , Tay FR , et al. Chlorhexidine preserves dentin bond in vitro. J Dent Res. 2007;86:90–4. 17189470 10.1177/154405910708600115 PMC2248723

[23] Epasinghe DJ , Yiu CK , Burrow MF , Tay FR , King NM . Effect of proanthocyanidin incorporation into dental adhesive resin on resin‐dentine bond strength. J Dent. 2012;40:173–80. 22155037 10.1016/j.jdent.2011.11.013

[24] Macedo FAA , Souza NO , Lemos MVS , De‐Paula DM , Santiago SL , Feitosa VP . Dentin bonding and physicochemical properties of adhesives incorporated with epigallocatechin‐3‐gallate. Odontology. 2019;107:23–8. 29796959 10.1007/s10266-018-0367-0

[25] Costa CAGA , Albuquerque NLG , Mendonça JS , Loguercio AD , Saboia VPA Santiago SL . Catechin‐based dentin pretreatment and the clinical performance of a universal adhesive: a two‐year randomized clinical trial. Oper Dent. 2020;45:473–83 32352353 10.2341/19-088-C

[26] Carrilho MR , Geraldeli S , Tay F , de Goes MF , Carvalho RM , Tjäderhane L , et al. In vivo preservation of the hybrid layer by chlorhexidine. J Dent Res. 2007;86:529–33. 17525352 10.1177/154405910708600608

[27] Ricci HA , Sanabe ME , de Souza Costa CA , Pashley DH , Hebling J . Chlorhexidine increases the longevity of in vivo resin‐dentin bonds. Eur J Oral Sci. 2010;118:411–16. 20662916 10.1111/j.1600-0722.2010.00754.x

[28] de Souza LC , Rodrigues NS , Cunha DA , Feitosa VP , Santiago SL , Reis A , et al. Two‐year clinical evaluation of proanthocyanidins added to a two‐step etch‐and‐rinse adhesive. J Dent. 2019,81:7–16. 30594631 10.1016/j.jdent.2018.12.012

[29] Demeule M , Brossard M , Pagé M , Gingras D , Béliveau R . Matrix metalloproteinase inhibition by green tea catechins. Biochim Biophys Acta. 2000;1478:51–60. 10719174 10.1016/s0167-4838(00)00009-1

[30] Garbisa S , Sartor L , Biggin, S . Salvato, B , Benelli, R , Albini A . Tumor gelatinases and invasion inhibited by the green tea flavanol epigallocatechin‐3‐gallate. Cancer. 2001;91:822–32. 11241252 10.1002/1097-0142(20010215)91:4<822::aid-cncr1070>3.0.co;2-g

[31] Hashimoto M , Ito S , Tay FR , Svizero NR , Sano H , Kaga M et al. Fluid movement across the resin‐dentin interface during and after bonding. J Dent Res. 2004;83:843–8. 15505233 10.1177/154405910408301104

[32] Pan Q , Xu Q , Boylan NJ , Lamb NW , Emmert D , Yang JC et al. Corticosteroid‐loaded biodegradable nanoparticles for prevention of corneal allograft rejection in rats. J Control Release. 2015;201:32–40. 25576786 10.1016/j.jconrel.2015.01.009 PMC6037178

[33] Silva AL , Rosalia RA , Varypataki E , Sibuea S , Ossendorp F , Jiskoot W . Poly‐(lactic‐co‐glycolic‐acid)‐based particulate vaccines: particle uptake by dendritic cells is a key parameter for immune activation. Vaccine. 2015;33:847–54. 25576216 10.1016/j.vaccine.2014.12.059

[34] Albuquerque NLG , Neri JR , Lemos MVS , Yamauti M , Sousa FFO , Santiago SL . Effect of polymeric microparticles loaded with catechin on the physicochemical properties of an adhesive system. Oper Dent. 2019,44:e202–e11. 30849016 10.2341/18-112-L

[35] Huang X , Brazel CS . On the importance and mechanisms of burst release in matrix‐controlled drug delivery systems. J Control Release. 2001;73:121–36. 11516493 10.1016/s0168-3659(01)00248-6

[36] Anusavice KJ , Zhang NZ , Shen C . Controlled release of chlorhexidine from UDMA‐TEGDMA resin. J Dent Res. 2006;85:950–4. 16998139 10.1177/154405910608501016 PMC2242729

[37] Ferracane JL . Hygroscopic and hydrolytic effects in dental polymer networks. Dent Mater. 2006;22:211–22. 16087225 10.1016/j.dental.2005.05.005

[38] Merdas I , Tcharkhtchi A , Thominette F , Verdu J , Dean K , Cook K . Water absorption by uncrosslinked polymers, networks and IPNs having medium to high polarity. Polymer. 2002;43:4619–25.

[39] Salz U , Zimmermann J , Zeuner F , Moszner N . Hydrolytic stability of self‐etching adhesive systems. J Adhes Dent. 2005;7:107–16. 16052759

[40] Malacarne J , Carvalho RM , de Goes MF , Svizero N , Pashley DH , Tay FR , et al. Water sorption/solubility of dental adhesive resins. Dent Mater. 2006;22:973–80. 16405987 10.1016/j.dental.2005.11.020

[41] Wambier L , Malaquias T , Wambier DS , Patzlaff RT , Bauer J , Loguercio AD , et al. Effects of prolonged light exposure times on water sorption, solubility and cross‐linking density of simplified etch‐and‐rinse adhesives. J Adhes Dent. 2014;16:229–34. 24847490 10.3290/j.jad.a32034

[42] Fabre HS , Fabre S , Cefaly DF , de Oliveira Carrilho MR , Garcia FC , Wang L . Water sorption and solubility of dentin bonding agents light‐cured with different light sources. J Dent. 2007;35:253–8. 17045723 10.1016/j.jdent.2006.09.002

[43] Danhier F , Ansorena E , Silva JM , Coco R , Le Breton A , Préat V . PLGA‐based nanoparticles: an overview of biomedical applications. J Control Release. 2012;161:505–22. 22353619 10.1016/j.jconrel.2012.01.043

[44] Hashimoto M , Nagano F , Endo K , Ohno H . A review: biodegradation of resin–dentin bonds. Jpn Dent Sci Rev. 2011;47:5–12

[45] Zanchi CH , Münchow EA , Ogliari FA , Chersoni S , Prati C , Demarco FF , et al. Development of experimental HEMA‐free three‐step adhesive system. J Dent. 2010; 38:503–8. 20302903 10.1016/j.jdent.2010.03.006

